# Impact of Photodynamic Therapy on Mast Cell-Associated Inflammation and Angiogenesis in Actinic Keratosis

**DOI:** 10.3390/cancers18183000

**Published:** 2026-09-16

**Authors:** Justyna Ceryn, Aleksandra Lesiak, Magdalena Ciążyńska, Olga Stasikowska-Kanicka, Karol Ciążyński, Joanna Narbutt

**Affiliations:** 1 Department of Dermatology, Paediatric Dermatology and Dermatological Oncology, Medical University of Lodz, 90-419 Lodz, Poland; lesiak_ola@interia.pl (A.L.); magdalena.ciazynska@umed.lodz.pl (M.C.); joanna.narbutt@umed.lodz.pl (J.N.); 2 International Doctoral School, Medical University of Lodz, 90-419 Lodz, Poland; 3 Laboratory of Autoinflammatory, Genetic and Rare Skin Disorders, Department of Dermatology, Pediatric Dermatology and Dermatological Oncology, Medical University of Lodz, 90-419 Lodz, Poland; 4 Chemotherapy Sub-Department, Specialist Oncological Hospital NU-MED sp. z o. o., 97-200 Tomaszów Mazowiecki, Poland; 5 One-Day Chemotherapy Department, Specialist Oncological Hospital NU-MED sp. z o. o., 97-200 Tomaszów Mazowiecki, Poland; 6 Department of Diagnostic Techniques in Pathomorphology, Medical University of Lodz, 90-419 Lodz, Poland; olga.stasikowska@umed.lodz.pl; 7 Institute of Applied Computer Science, Lodz University of Technology, 90-419 Lodz, Poland; karol.ciazynski@gmail.com

**Keywords:** actinic keratosis, photodynamic therapy, mast cells, mast cell tryptase, angiogenesis, CD31

## Abstract

**Background/Objectives:** Actinic keratosis (AK) is a common ultraviolet-induced intraepidermal neoplasm with a recognized potential to progress to cutaneous squamous cell carcinoma (cSCC). Although photodynamic therapy (PDT) is an established field-directed treatment for AK, its effects on the inflammatory and angiogenic tumor microenvironment, particularly regarding mast cell (MC) infiltration and microvascular remodeling, remain incompletely understood. This study aimed to investigate the impact of PDT on mast cell (MC) count and CD31-associated microvessel density (MVD) in AK lesions. **Methods:** In this prospective single-center study, 31 patients with histopathologically confirmed AK underwent three sessions of 5-aminolevulinic acid-based PDT. Each patient contributed samples of clinically uninvolved skin, the target AK lesion before PDT, and the same AK lesion after PDT. Repeated tissue measurements were analyzed using the Friedman test followed by exact Wilcoxon signed-rank tests with Holm correction. Associations between MC count and MVD were assessed using Spearman rank correlations. Clinical and dermoscopic grades were analyzed as paired ordinal outcomes. **Results:** Significant clinical and dermoscopic improvement was observed in the majority of patients following PDT, with complete lesion clearance achieved in 17 of 31 patients (54.84%). Both MC count and MVD were significantly higher in AK lesions compared with clinically uninvolved skin (*p* < 0.05). PDT resulted in a significant reduction in both parameters (*p* < 0.05), although post-treatment levels remained elevated relative to clinically uninvolved skin. MC count and MVD were positively correlated both before and after PDT, with a stronger association observed after treatment. **Conclusions:** PDT demonstrates not only cytotoxic effects against dysplastic keratinocytes but also may significantly modulate the inflammatory and angiogenic microenvironment in AK. The persistent correlation between MC infiltration and neovascularization after therapy may reflect incomplete normalization of the local microenvironment despite clinical improvement. These findings suggest that MC count and MVD may merit further evaluation as adjunctive biomarkers of response in PDT-treated AK.

## Simple Summary

Actinic keratosis (AK) is a common lesion caused by chronic ultraviolet exposure and is regarded as a precursor to cutaneous squamous cell carcinoma. Photodynamic therapy (PDT) is an established treatment option for AK, but its effects on the local cutaneous microenvironment remain insufficiently characterized. In this study, we assessed changes in mast cell count and microvessel density in AK lesions treated with PDT, comparing pre- and post-treatment specimens with adjacent clinically uninvolved skin. PDT was associated with histological improvement of the lesions and with a reduction in both mast cell infiltration and microvascular density, although neither parameter returned completely to the level observed in clinically uninvolved skin. These findings suggest that PDT may induce partial remodeling of the inflammatory and vascular microenvironment in AK, warranting further validation in larger cohorts with longer follow-up.

## 1. Introduction

Actinic keratosis (AK) is a common intraepidermal proliferative disorder characterized by atypical keratinocyte expansion restricted to the epidermis, representing a well-established precursor to cutaneous squamous cell carcinoma (cSCC). AK shows a marked predilection for older individuals, with epidemiological studies estimating its prevalence to approach 25% in the general population. The incidence notably increases with age, reaching 14.57% among individuals over 80 years old [1,2]. Clinically, AK manifests as erythematous, scaly, or hyperkeratotic macules or plaques, most commonly located in chronically sun-exposed regions such as the face, scalp, forearms, and dorsal hands [3]. Chronic ultraviolet radiation (UVR) is the principal etiological factor, and individuals with Fitzpatrick skin types I and II are particularly susceptible [4]. Although AK is considered a preinvasive lesion, the risk of progression to invasive squamous cell carcinoma (iSCC) is well documented and varies depending on lesion characteristics and patient history, with annual conversion rates estimated between 0.025% and 16% [5,6].

Importantly, AKs often arise within a broader context of “field cancerization” (FC), referring to areas of skin exhibiting subclinical UV-induced genetic damage, which predispose to the development of multiple neoplastic lesions [7]. This concept highlights the importance of therapeutic approaches that target not only clinically visible lesions but also the adjacent photodamaged skin, which may contain subclinical dysplastic alterations.

Management of AK typically involves two therapeutic approaches: lesion-directed interventions targeting isolated lesions, and field-directed therapies addressing both clinical and subclinical lesions within FC [3,8]. Lesion-oriented approaches are appropriate in cases presenting with fewer than five AKs, limited photodamage, and low individual risk for non-melanoma skin cancers (NMSCs), whereas field-directed strategies are recommended for patients with extensive actinic damage, multiple lesions, or high oncological risk. Both therapeutic modalities encompass topical agents, including imiquimod, 5-fluorouracil, and diclofenac, as well as procedural interventions such as cryotherapy, photodynamic therapy (PDT), chemical peeling, laser ablation, electrocoagulation, and curettage [3].

PDT, either in its conventional (c-PDT) or daylight (dl-PDT) form, has emerged as an effective treatment modality for widespread AK. Current European (2024) [3] and American (2021) [8] guidelines consistently endorse the use of PDT based on its efficacy and favorable cosmetic outcomes. Among available protocols, conventional 5-ALA-based PDT (5-aminolevulinic acid-based PDT) using a 10% 5-aminolevulinic acid (5-ALA) nanoemulsion gel and red light exposure has demonstrated the highest clearance rates estimating 90% following a single session [8,9,10,11,12,13].

Mast cells (MCs) are multifunctional, tissue-resident effector cells best known for their roles in allergic reactions and in host defense mechanisms against various pathogens [14]. Beyond these classical functions, MCs contribute importantly to tissue remodeling, wound healing, and the maintenance of vascular homeostasis. In recent years, mounting evidence has drawn attention to the involvement of MCs in carcinogenesis, including cutaneous malignancies [15]. Chronic inflammation is a well-established driver of tumor development, and this has prompted investigation into the immune components of the tumor microenvironment. Within this context, MCs have emerged as key modulators that link persistent inflammation to neoplastic progression [15].

Neovascularization plays a critical role in tumor development and progression, serving as both a hallmark of malignancy and a marker of chronic inflammation in AK. Inflammatory mediators and MCs contribute significantly to the formation of new microvessels within AK lesions, facilitating the transition from pre-neoplastic to invasive stages. Studies have demonstrated a marked increase in microvascular density accompanying the progression from AK to cSCC, underscoring the prognostic relevance of angiogenic activity [16,17]. Among molecular markers of angiogenesis, CD31 (Platelet Endothelial Cell Adhesion Molecule-1, PECAM-1) is particularly valuable for the assessment of endothelial cell presence and vessel density, and the quantification of neoangiogenesis [18].

Although PDT is widely recognized as an effective field-directed therapy for AK, its immunological and vascular effects, particularly those involving mast cell-mediated responses and microvascular remodeling, are still not fully characterized. A deeper exploration of these pathways may offer new insights into the multifaceted action of PDT, extending beyond its direct cytotoxicity and highlighting its potential role in modulating inflammation and neovascularization within the carcinogenic field.

The aim of our study was to evaluate MC count and microvessel density (MVD) in clinically uninvolved skin and in AK lesions before and after PDT. MC infiltration was assessed by immunohistochemical staining of skin biopsy specimens with a monoclonal antibody against mast cell tryptase. Angiogenesis was evaluated by immunohistochemical staining with monoclonal antibodies against CD31, a well-established endothelial cell marker.

To the best of our knowledge, this work provides the first prospective clinical data on the effects of PDT on MC-related changes and associated angiogenic markers in premalignant AK lesions.

## 2. Materials and Methods

### 2.1. Study Population

This single-center, prospective clinical study was conducted at the Department of Dermatology, Paediatric Dermatology, and Oncology, Medical University of Lodz, and enrolled adult patients with histologically confirmed AK. The study protocol was reviewed and approved by the Bioethics Committee of the Medical University of Lodz. Written informed consents were obtained from all participants prior to inclusion. All eligible patients available during the study period were included—no formal a priori sample size calculation was performed. Key eligibility criteria included histopathologically confirmed and previously untreated AK. Key exclusion criteria included active malignancy, including NMSCs and malignant melanoma (MM). A complete overview of inclusion and exclusion criteria is provided in Table 1.

### 2.2. Study Design and Assessments (Clinical, Dermoscopic, Histopathological and Immunohistochemical Evaluation)

The study design comprised three PDT sessions administered at four-week intervals. Clinical and dermoscopic evaluations were performed at baseline, prior to each photodynamic therapy session, and at the final follow-up visit eight weeks after the last treatment. Standardized photographic and dermoscopic documentation was obtained at all time points to ensure reliable and consistent monitoring of lesion dynamics. Lesions were identified as AK based on characteristic clinical features, including erythematous, scaly, and keratotic plaques with a rough, sandpaper-like surface texture [19]. Dermoscopic diagnosis of AK was based on established morphological criteria, including a pink-to-reddish pseudonetwork encircling follicular openings, presence of yellowish-white scaling, thin, wavy vascular structures, and follicular keratotic plugs—collectively referred to as the characteristic “strawberry pattern” [19,20]. In addition, the presence of the “rosette sign”, four whitish points arranged in a clover-like formation around a follicular opening, was considered diagnostic [21].

The severity of AK was graded clinically using Olsen’s classification system (grades 1–3), as outlined in Table 2 [22]. To improve diagnostic accuracy, we employed the dermoscopic severity scale originally developed by Zalaudek et al., incorporating minor modifications to better capture the morphological heterogeneity of AK lesions within areas of FC [23,24,25]. This included the addition of intermediate categories (I/II and II/III) to represent transitional morphologies not fully captured by the classical three-tier scale (grades I–III), thereby allowing for a more nuanced stratification of lesion severity (Table 2).

Baseline punch biopsies (≥4 mm in diameter) were obtained from both the clinically diagnosed AK lesions and clinically uninvolved skin (control group). These samples were collected before the initiation of PDT in order to confirm the histopathological diagnosis and enable subsequent immunohistochemical analyses. At the final follow-up visit, an additional biopsy was obtained from the site of the previously treated AK.

Prior to PDT, the target AK lesions were thoroughly cleansed, including mechanical removal of surface scales and crusts, followed by degreasing. Then, a thin layer (~1 mm) of 10% 5-ALA gel (Ameluz^®^, Biofrontera, Inc., Wakefield, MA, USA) was carefully applied to the entire surface of each AK lesion as well as to approximately 5 mm of the surrounding clinically uninvolved skin. The gel was allowed to air dry for approximately 10 min before the treated area was covered with a non-absorbent, occlusive dressing for a standardized incubation period of 3 h. Upon completion of incubation, the dressing was carefully removed, and the skin surface was gently rinsed with 0.9% sodium chloride solution. Light activation was then performed using a red-light source (Treviolux; MEDlight GmbH, Herford, Germany, pulsed red light, 630 nm) delivering a total radiant exposure of 37 J/cm^2^ at an irradiance of 40 mW/cm^2^, with an illumination time of approximately 15.42 min per treatment site. The standard distance between the light source and the irradiated skin surface was maintained at 5–8 cm, as per device specifications.

Following standard histopathological protocols, all biopsy specimens were promptly fixed in 10% neutral buffered formalin to preserve tissue architecture. Subsequently, samples underwent routine processing and paraffin embedding, followed by microtome sectioning and staining with hematoxylin and eosin (H&E). Histopathological evaluation was performed in accordance with contemporary diagnostic criteria for AK and related epidermal alterations.

Immunohistochemical staining was carried out following a standardized protocol. Tissue sections, cut at 3 μm thickness, were initially deparaffinized in xylene and rehydrated through a descending ethanol gradient. Antigen retrieval was performed by microwave heating in Target Retrieval Solution (TRS, pH 9.0; Dako, Glostrup, Denmark) for 30 min. Endogenous peroxidase activity was quenched with 0.3% hydrogen peroxide in methanol for 30 min. Following rinsing in TBS, the slides were incubated for 30 min with primary mouse monoclonal antibodies targeting MC tryptase (mouse monoclonal Ab; clone AA1; No kat M5072; dilution 1:200; Dako, Glostrup, Denmark) and CD31 (mouse monoclonal Ab; clone QBEnd 10; No kat IR604; RTU; Dako, Glostrup, Denmark).

After washing, an adequate EnVision-HRP detection system (Dako, Carpinteria, CA, USA) was applied, with 3,3′-diaminobenzidine serving as the chromogen. Slides were then counterstained with Mayer’s hematoxylin, followed by sequential washing, dehydration, clearing in xylene, and coverslipping. For negative controls, the same staining protocol was applied with substitution of the primary antibody by an appropriate antibody diluent.

The number of MCs, identified by granular, cytoplasmic tryptase staining, was quantified by two independent observers across all viable high-power fields (HPFs) available per tissue section. The number of fields analyzed varied between 3 and 10 HPFs depending on the preserved area of each individual specimen; however, each tissue section was assessed in its entirety. The MC count was expressed as the mean number of immunopositive cells per HPF (field area = 0.029 mm^2^).

MVD was assessed using the “hotspot” method by counting CD31-positive luminal structures in 3 to 7 HPFs with the highest vascular density, while low-density areas were excluded. Each slide was evaluated twice, independently, by two experienced observers. In cases of disagreement, a joint re-evaluation was performed to achieve consensus.

### 2.3. Statistical Analysis

Statistical analyses were performed at patient level. The study included 31 independent patients, each contributing three related tissue samples: clinically uninvolved skin, the target AK lesion before PDT, and the same AK lesion after PDT. Quantitative variables were summarized using means and standard deviations and medians with interquartile ranges, as appropriate. Categorical variables were summarized as numbers and percentages. Normality of the within-patient differences was assessed using the Shapiro–Wilk test and Q–Q plots. Because the normality assumption was not consistently satisfied, differences among the three related tissue states were evaluated separately for MC count and MVD using the Friedman test. Significant omnibus results were followed by exact two-sided Wilcoxon signed-rank tests for the following pairwise contrasts AK before PDT versus uninvolved skin, AK after PDT versus AK before PDT, and AK after PDT versus clinically uninvolved skin. Holm’s procedure was applied separately within each biomarker family. Kendall’s W was reported as the effect size for the Friedman test. Pairwise effect sizes were reported as matched-pairs rank-biserial correlations. Median within-patient differences were also calculated. Ninety-five percent confidence intervals for Kendall’s W, rank-biserial correlations, and median paired differences were obtained using 10,000 patient-level bootstrap resamples. Associations between MC count and MVD were evaluated separately in clinically uninvolved skin, AK before PDT, and AK after PDT using Spearman rank correlation. Numerical two-sided *p*-values were obtained using 100,000 patient-level permutations. Holm’s procedure was applied across the three tissue-specific correlation analyses, and 95% confidence intervals were calculated using 10,000 patient-level bootstrap resamples. Changes in Olsen and dermoscopic grades were analyzed as paired ordinal outcomes using exact two-sided Wilcoxon signed-rank tests. Holm’s procedure was applied across the two comparisons. Response proportions were reported with exact Clopper–Pearson 95% confidence intervals. All tests were two-sided, with α = 0.05.

## 3. Results

### 3.1. Study Group Clinical and Dermoscopic Evaluation

**Figure 1:**
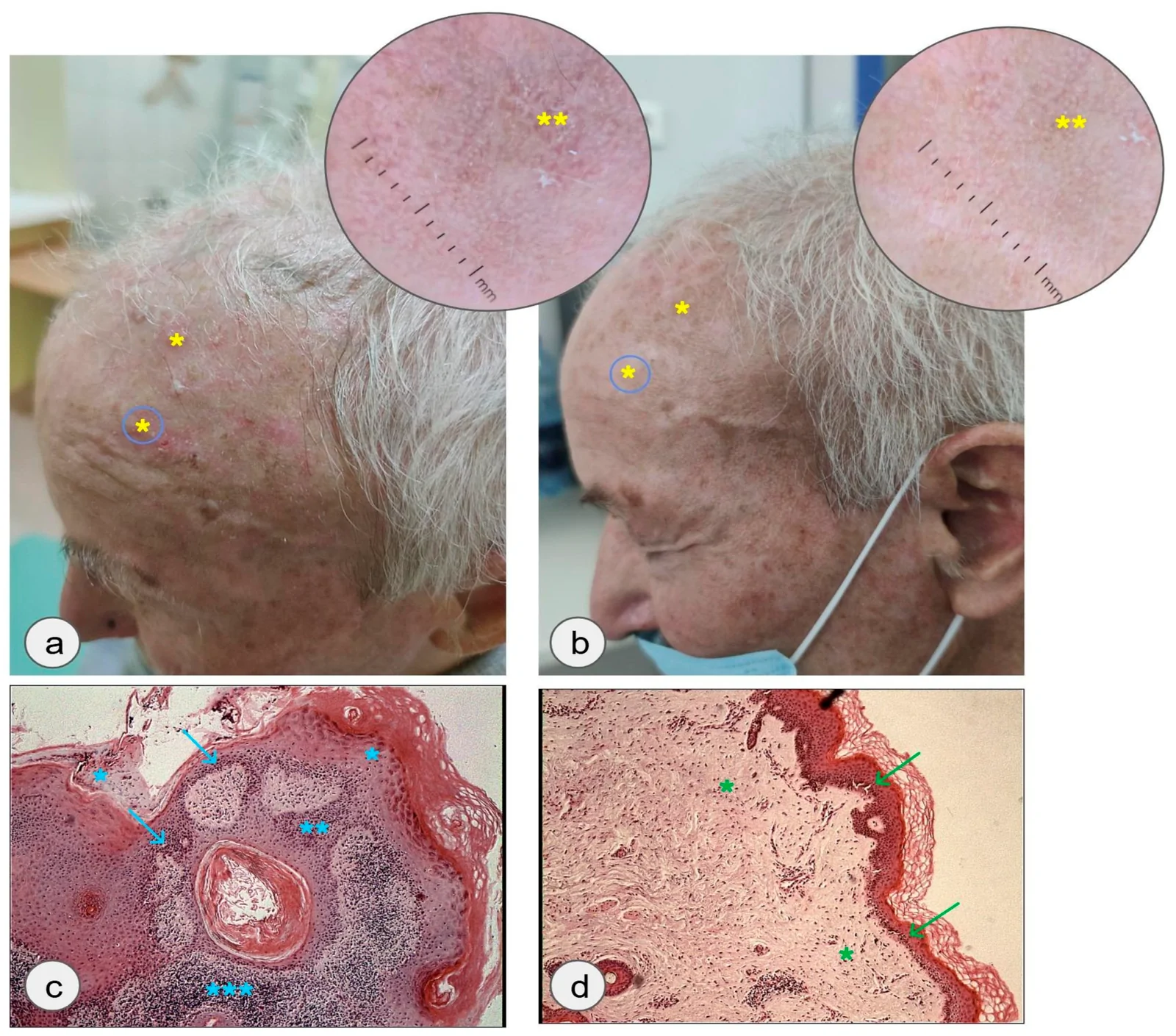
Clinical and dermoscopic evaluation of actinic keratosis (AK) lesions before (**a**) and after (**b**) the photodynamic therapy (PDT). Panel (**a**) presents the baseline clinical image of the scalp with erythematous, scaly AK lesions (yellow asterisks *) within a photodamaged field. A target lesion marked with a blue circle was selected for dermoscopic follow-up. The magnified dermoscopic view (**top** inset, (**a**)) demonstrates a disrupted pseudo-network with white-yellowish scaling, dotted vessels, and follicular keratotic plugs (yellow asterisks **) typical of AK. Panel (**b**) presents the same area eight weeks after the final PDT session. Clinically, marked improvement is evident, with resolution of hyperkeratosis and erythema in the treated area (yellow asterisks *). The target lesion (blue circle) shows flattening and repigmentation. Dermoscopically (**top** inset, (**b**)), the previously observed AK-specific structures have regressed; the pseudo-network is less pronounced, keratin plugs have disappeared, and scaling is minimal (yellow asterisks **). These findings indicate a successful treatment response at both macroscopic and dermoscopic levels. Histological analysis of an AK lesion before (**c**) and after (**d**) PDT, showing morphological changes following treatment (hematoxylin and eosin (H&E) staining; magnification ×100). Panel (**c**)—H&E staining reveals marked epidermal atypia (blue arrows) characterized by nuclear pleomorphism and loss of polarity, disordered keratinocyte maturation, hyperkeratosis, prominent focal parakeratosis (blue asterisks *) and irregular acanthosis (blue asterisks **). The basal and suprabasal layers show pronounced keratinocyte atypia, with a dense lymphocytic infiltrate in the upper dermis (blue asterisk ***). Panel (**d**)—the histological section demonstrates partial re-epithelialization and restoration of epidermal architecture. A reduction in keratinocyte atypia and reorganization of the epidermal layers is evident (green arrows). The stratum corneum appears more compact and orthokeratotic. The inflammatory infiltrate in the papillary dermis is markedly diminished, with reduced cellular density (green asterisks *).

A total of 31 adult patients diagnosed with AK were prospectively enrolled in the study (Table 3 summarizes the patient characteristics). All 31 participants successfully completed the entire research protocol, including all scheduled PDT treatment sessions, clinical and dermoscopic evaluations, as well as both baseline and post-treatment histopathological and immunohistochemical assessments. The majority of AK lesions were localized on the scalp (*n* = 17; 54.84%), with additional involvement of the face, forearms, and trunk. Prior to initiating PDT, the diagnosis of AK was histopathologically confirmed in each patient through skin biopsy. Majority of participants exhibited clinical and dermoscopic improvement following the PDT protocol (Figure 1). Complete clinical clearance of AK lesions was observed in 17 patients (54.84%), whereas the remaining 14 individuals (45.16%) demonstrated a reduction in lesion severity to Olsen grade 1. Importantly, no grade 2 or 3 lesions were present post-treatment. Dermoscopic evaluation revealed complete lesion resolution in 8 patients (25.81%), with 16 (51.61%) downgraded to grade I and 7 (22.58%) to grade II. As with clinical grading, no cases of grade III severity were observed after the treatment course.

Adverse events (AEs) were reported in 18 patients (58.06%) during PDT sessions. The most frequent events, observed in 16 patients (51.61%), were mild local reactions, mainly burning or pinching sensations during or after the procedure, which required temporary measures such as interruption of irradiation or cooling with air. Two patients (6.45%) developed severe local AEs in the form of burns at the treated site within several days after completion of the initial PDT session, requiring escalation of treatment with topical and systemic corticosteroids and oral antihistamines. These reactions resolved within a few days after the initiation of symptomatic treatment, and subsequent PDT sessions were completed without modification of the treatment protocol. In addition, both patients did not adhere to post-procedure photoprotection recommendations and exposed the treated areas to ultraviolet radiation shortly after illumination, which likely intensified the local phototoxic response. Both patients had advanced AK lesions (Olsen grade 3 and dermoscopic severity scale III), and complete clinical clearance of the treated lesions was ultimately achieved in both cases. Notably, these findings suggest that greater lesion severity may be associated with an increased risk of pronounced post-PDT local reactions and may also influence the subsequent clinical course; however, this requires further validation.

### 3.2. Mast Cell Count and Microvessel Density—Immunohistochemical Evaluation and Statistical Analysis

**Figure 2:**
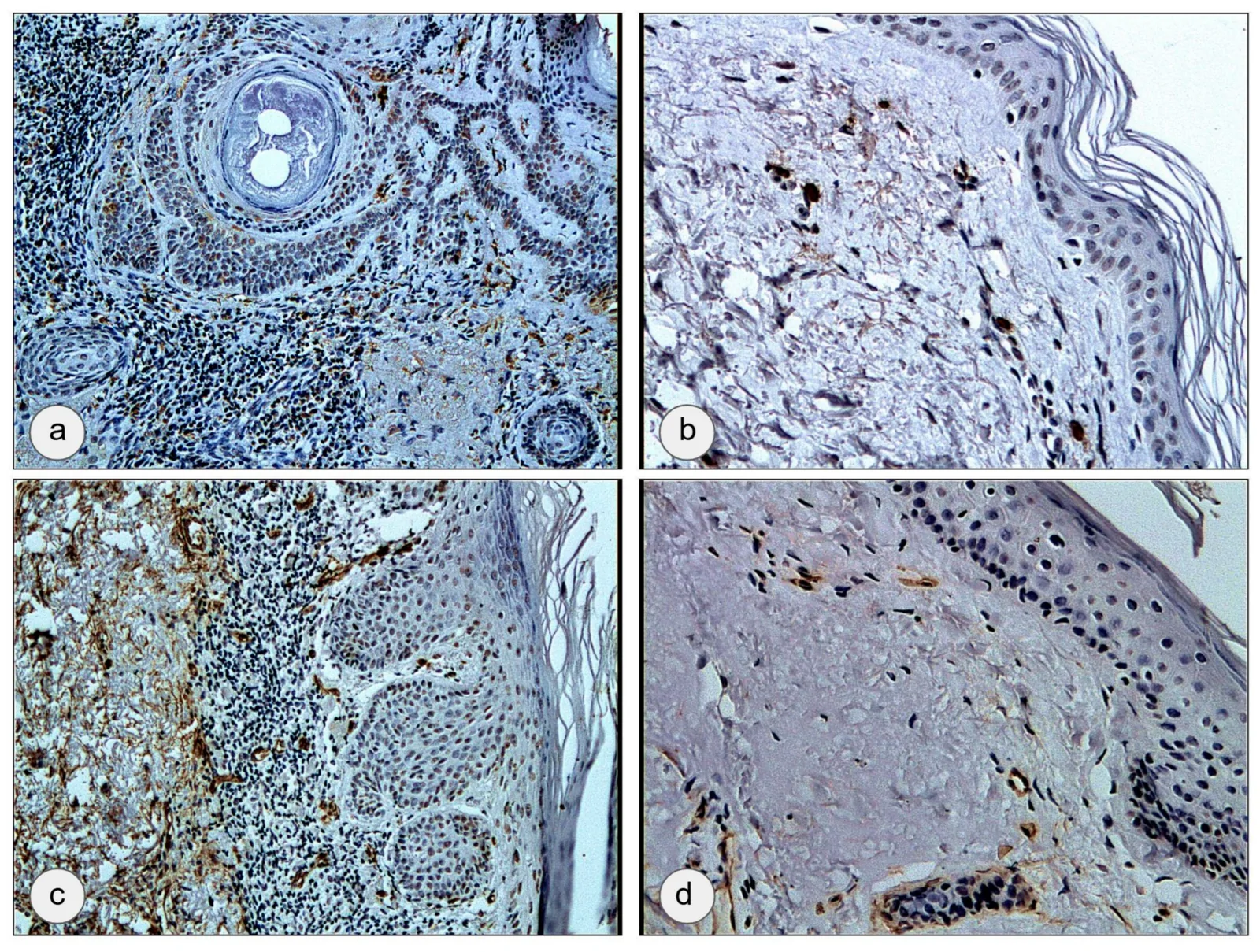
Immunoexpression of selected markers in an actinic keratosis (AK) lesion before and after photodynamic therapy (PDT). Mast cell tryptase immunostaining of an AK lesion before (**a**) and after (**b**) PDT, showing a lower number of tryptase-positive cells after treatment (immunohistochemistry (IHC), strong cytoplasmic and weak extracellular staining; original magnification ×100 and ×200, respectively); CD31 immunostaining of an AK lesion before (**c**) and after (**d**) PDT, showing reduced microvessel density following treatment (IHC, original magnification ×150 and ×200, respectively).

**Figure 3:**
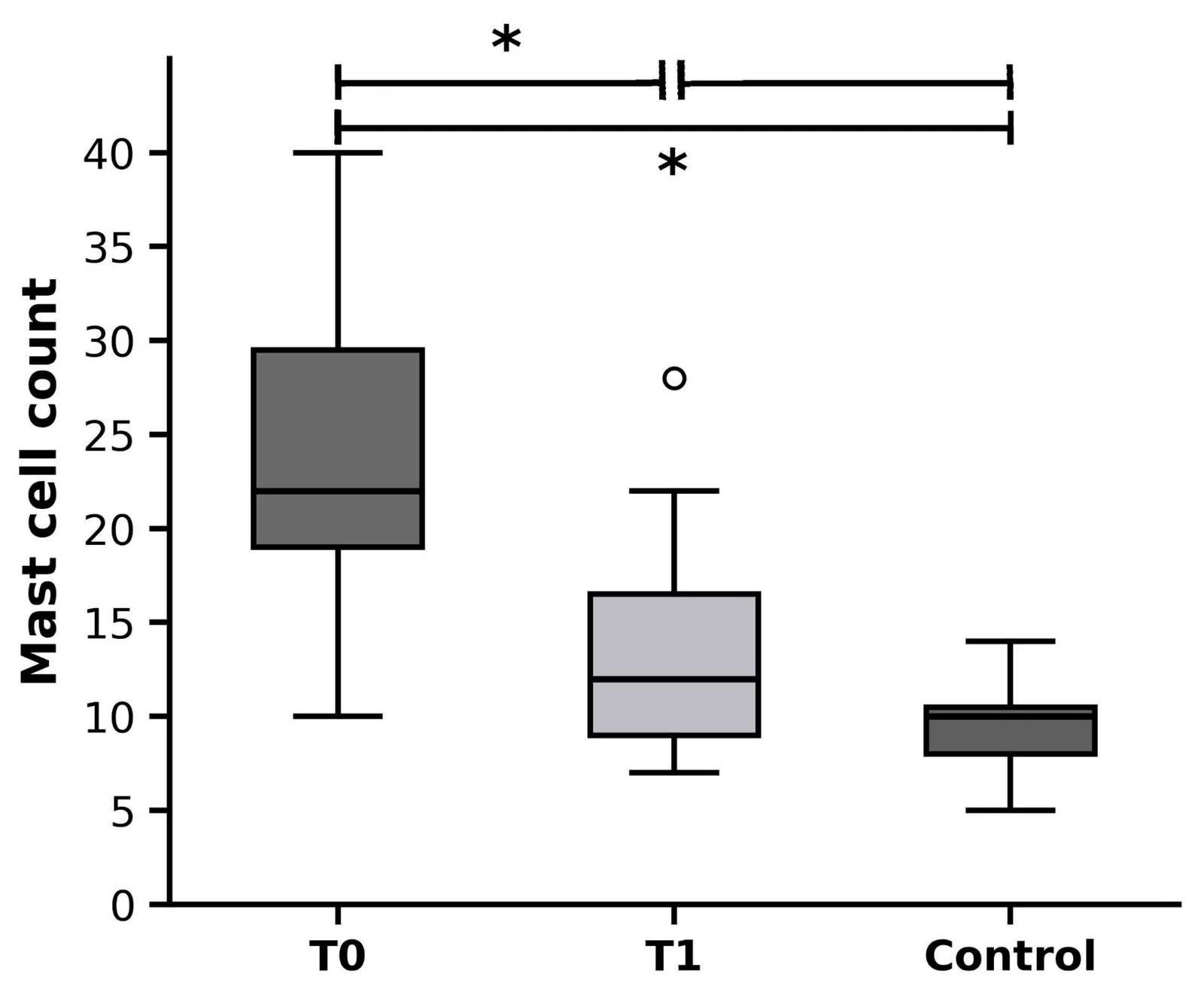
Boxplot representation of mast cell (MC) count across the study groups; MC count was significantly higher before photodynamic therapy (PDT) (T0) than in clinically uninvolved skin (Control) and decreased significantly following PDT (T1). Post-treatment MC count nevertheless remained higher than in Control (*p* < 0.05); * *p* < 0.05.

MC count varied significantly across the three analyzed tissue states: clinically uninvolved skin (Control), AK prior to PDT, and AK following PDT (Figure 2). The lowest MC count was observed in clinically uninvolved skin (mean 9.26 ± 2.25), whereas untreated AK lesions exhibited a marked increase in MC infiltration (mean 24.13 ± 7.38). Post-therapeutic evaluation revealed a substantial reduction in MC count (mean 13.29 ± 5.25), although values did not fully normalize to baseline levels observed in clinically uninvolved skin (control). Pairwise exact Wilcoxon signed-rank tests confirmed that MC count was significantly higher in AK before PDT than in matched clinically uninvolved skin (raw *p* = 9.313 × 10^−10^; Holm-adjusted *p* = 2.794 × 10^−9^), significantly lower after PDT than before treatment (raw *p* = 9.313 × 10^−10^; Holm-adjusted *p* = 2.794 × 10^−9^), and significantly higher after PDT than in clinically uninvolved skin (raw and Holm-adjusted *p* = 0.0008607). Boxplot distributions for each group are presented in Figure 3. When analyzed by Olsen grade, lesions classified as Olsen grade 3 showed numerically higher MC count compared with grade 1–2 lesions; however, these differences were not statistically significant.

MC count differed significantly among clinically uninvolved skin, AK before PDT, and AK after PDT (Friedman χ^2^(2) = 52.323, *p* = 4.35 × 10^−12^; Kendall’s W = 0.844, 95% CI 0.771–0.940). Median MC count was 10.0 (IQR 8.0–10.5) in clinically uninvolved skin, 22.0 (IQR 19.0–29.5) in AK before PDT, and 12.0 (IQR 9.0–16.5) after PDT (Figure 3).

MC count was higher in AK before PDT than in clinically uninvolved skin (exact *p* = 9.313 × 10^−10^; Holm-adjusted *p* = 2.794 × 10^−9^). It decreased after PDT compared with pretreatment values (exact *p* = 9.313 × 10^−10^; Holm-adjusted *p* = 2.794 × 10^−9^) but remained higher than in clinically uninvolved skin (exact and Holm-adjusted *p* = 0.0008607). Pairwise effect sizes and confidence intervals are presented in Table 4.

**Figure 4:**
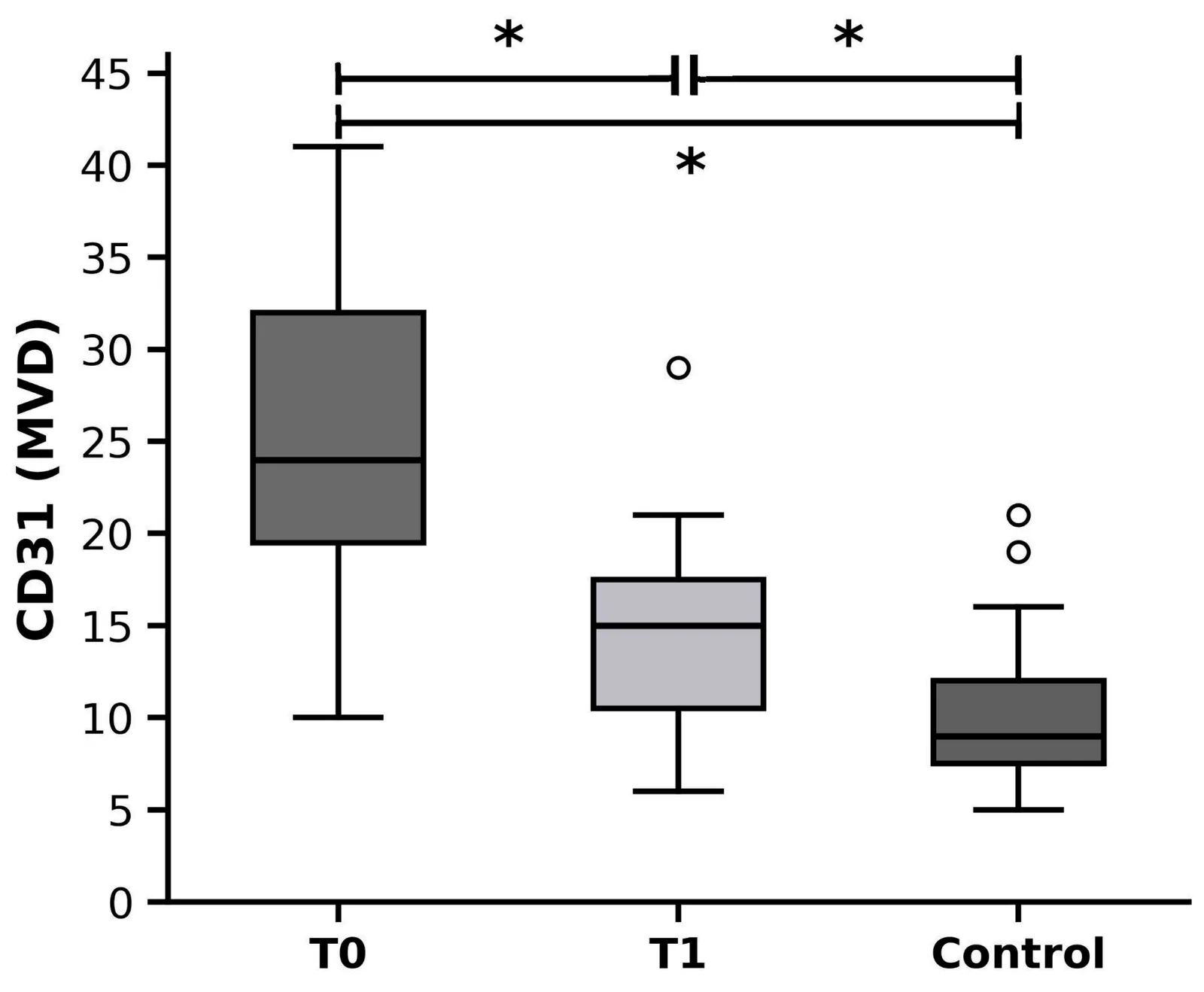
Boxplot representation of microvessel density (MVD, CD31-positive cells) across the study groups; Post-treatment analysis revealed a statistically significant reduction in MVD (mean 14.55 ± 5.18; *p* < 0.05); however, values remained elevated compared to clinically uninvolved skin (Control) (*p* < 0.05); * *p* < 0.05. **Abbreviations:** T0, before PDT; T1, after PDT; Control, clinically uninvolved skin.

MVD also differed significantly among the three tissue states (Friedman χ^2^(2) = 47.617, *p* = 4.57 × 10^−11^; Kendall’s W = 0.768, 95% CI 0.668–0.883). Median MVD was 9.0 (IQR 7.0–12.0) in clinically uninvolved skin, 24.0 (IQR 19.5–32.0) in AK before PDT, and 13.0 (IQR 10.0–17.0) after PDT (Figure 4).

MVD was higher in AK before PDT than in clinically uninvolved skin (exact *p* = 9.313 × 10^−10^; Holm-adjusted *p* = 2.794 × 10^−9^). It decreased after PDT compared with pretreatment values (exact *p* = 1.788 × 10^−7^; Holm-adjusted *p* = 3.576 × 10^−7^) but remained higher than in clinically uninvolved skin (exact and Holm-adjusted *p* = 0.0005826). Pairwise effect sizes and confidence intervals are presented in Table 4.

MC count and MVD were positively correlated in AK before PDT (*n* = 31; Spearman’s ρ = 0.630, 95% CI 0.325–0.822; raw *p* = 0.000270; Holm-adjusted *p* = 0.000540) and after PDT (*n* = 31; ρ = 0.736, 95% CI 0.476–0.878; raw *p* = 0.000010; Holm-adjusted *p* = 0.000030). In clinically uninvolved skin, the correlation did not reach statistical significance after correction (*n* = 31; ρ = 0.354, 95% CI −0.004 to 0.667; raw and Holm-adjusted *p* = 0.051849). These findings demonstrate associations between MC count and MVD in AK lesions but do not establish directionality, functional mediation, or causality.

### 3.3. Olsen Grade and Dermoscopic Grade—Statistical Analysis

Olsen grade improved in 29 of 31 patients (93.5%; exact 95% CI 78.6–99.2%), remained unchanged in two patients, and worsened in none. Complete clinical clearance, defined as Olsen grade 0, was observed in 17 patients (54.8%; exact 95% CI 36.0–72.7%). Fourteen patients had residual Olsen grade 1 after treatment, and no grade 2 or 3 lesions were present at follow-up. The median paired change was −1 Olsen grade (95% bootstrap CI −2 to −1). The paired change was statistically significant (Wilcoxon T = 0; raw *p* = 3.73 × 10^−9^; Holm-adjusted *p* = 3.73 × 10^−9^; matched-pairs rank-biserial correlation = −1.000).

Dermoscopic grade improved in 30 of 31 patients (96.8%; exact 95% CI 83.3–99.9%), remained unchanged in one patient, and worsened in none. Complete dermoscopic resolution was observed in 8 patients (25.8%; exact 95% CI 11.9–44.6%). At follow-up, 16 patients had dermoscopic grade I and 7 had grade II; no grade III lesions were observed. The median paired change was −1 category (95% bootstrap CI −1.5 to −1). The paired change was statistically significant (Wilcoxon T = 0; raw *p* = 1.86 × 10^−9^; Holm-adjusted *p* = 3.73 × 10^−9^; matched-pair rank-biserial correlation = −1.000).

## 4. Discussion

In the early stages of skin carcinogenesis, such as in AK, chronic inflammatory stimuli create a microenvironment that promotes MC activation and accumulation. Repeated UV damage and other carcinogenic insults lead to ongoing tissue injury and repair, which not only drives local proliferation of resident MCs but also promotes the recruitment of circulating MC precursors to sites of dysplasia [16]. The presence of MCs in these premalignant lesions suggests that they may contribute to early events in tumor development. Notably, only a few studies have explicitly documented MC infiltration in cutaneous neoplasms. These reports include observations of MCs at the periphery or within the stroma of NMSCs, such as cSCC and basal cell carcinoma (BCC), as well as in MM [19,20,21,26,27,28,29,30]. Despite their limited number, these studies consistently highlight the multifaceted role of MCs in tumor biology.

Upon activation, MCs release a broad array of pro-inflammatory and pro-angiogenic mediators, including histamine, tryptase, and growth factors like vascular endothelial growth factor (VEGF), which can profoundly reshape the tumor microenvironment [31]. Through these mediators, MCs help induce new blood vessel formation and extracellular matrix (ECM) degradation, processes that are critical for tumor expansion, invasion, and metastasis [32]. MCs are also increasingly recognized for their immunomodulatory impact on tumor microenvironment [33]. They contribute to the establishment of local immune tolerance, for example by promoting the recruitment and induction of regulatory T cells (Tregs), thereby dampening anti-tumor immune responses and facilitating tumor immune evasion [20]. In addition, MCs can exert direct mitogenic effects on neoplastic cells, further supporting tumor growth and progression [33]. In conclusion, from the earliest precancerous changes in the skin (such as those in AK) through to invasive malignancies, MCs appear to be influential orchestrators in the interplay between chronic inflammation and skin cancer development, driving several mechanisms that promote tumor survival and expansion [32].

In this study, we investigated the impact of PDT on MC infiltration and angiogenesis in AK lesions, integrating clinical, dermoscopic, and immunohistochemical assessments. Our data confirms that AK is characterized by a substantial accumulation of MCs and increased MVD compared with clinically uninvolved skin. This is consistent with the broader view of AK as a prototypical UV-induced intraepidermal neoplasia that develops in a chronically inflamed and pro-angiogenic microenvironment. Our key finding was that both MC count and MVD were markedly reduced in treated AK lesions following PDT.

Clinically and dermoscopically, all patients demonstrated improvement after PDT, with more than half achieving complete clearance or downgrading to Olsen grade 1 and no residual grade 2–3 lesions at follow-up. These findings suggest that, in addition to its effects on atypical keratinocytes, PDT may be associated with modulation of the local tissue microenvironment, including reductions in inflammatory and vascular components accompanying clinical improvement.

An important observation of our study is the positive correlation between MC count and MVD at both time points, which was stronger after PDT than before treatment. These data are consistent with a potential relationship between MCs and angiogenesis in AK, although they do not allow conclusions regarding direct mechanistic involvement.

After PDT, both MC count and MVD showed a significant reduction relative to baseline; however, both parameters remained significantly elevated compared with clinically uninvolved skin. From a therapeutic perspective, our findings suggest that PDT was accompanied by a reduction in both MC count and MVD in AK lesions. Nevertheless, neither parameter fully returned to the lower levels observed in clinically uninvolved skin, indicating only partial normalization after treatment. The continued association between MC count and MVD after PDT may reflect a persistent link between inflammatory and vascular components of the lesion microenvironment but further studies are needed to fully understand the residual biologic activity within the treated lesion, the potential recurrence risk, or the need for repeated treatment.

Rosin et al. [34] conducted the only known experimental study to date examining PDT’s impact on MCs and MVD in premalignant lesions, using a 4-nitroquinoline-1-oxide(4NQO)-induced oral carcinogenesis rat model [34]. They reported comparable observation that after 5-ALA-PDT, MC density remained strongly correlated with MVD. However, in their study, PDT also triggered an acute inflammatory response, with a marked increase in MC degranulation and early microvascular dilation within 6–24 h after treatment. Rosin et al. [34] interpreted the persistent post-PDT MC activity as a possible contributor to “reparative” angiogenesis, which might, under some circumstances, promote tumor progression in lesions that do not fully regress after therapy. In contrast, our prospective human study on AK uniquely integrated immunohistochemical analyses with clinical and dermoscopic outcomes and demonstrated a significant reduction in both MC count and CD31-defined MVD in treated AK lesions two months after PDT. By selecting this later point, we aimed to reduce the influence of acute post-treatment inflammation and tissue repair on the analysis. At this stage, the tissue likely reflected a more stable post-therapeutic state rather than the immediate reactive phase seen shortly after PDT. Overall, this human data may suggest partial normalization of the inflammatory and vascular microenvironment after effective PDT, although they do not allow conclusions about mechanism, residual pro-angiogenic activity, or recurrence risk. To our knowledge, this is the first prospective clinical study in humans to link changes in MC populations and angiogenesis markers with both clinical and dermoscopic response after PDT in AK, underscoring the novelty and translational relevance of these observations.

It is important to note that MCs may play context-dependent roles in tumor biology. While many studies have linked MCs to tumor-promoting processes such as immunosuppression, angiogenesis, matrix remodeling, and growth factor release, other reports suggest that they might also contribute to anti-tumor responses in certain settings [33]. This possibility has been discussed in the context of immune-modulating therapies, for example imiquimod, a Toll-like receptor 7 (TLR7) agonist, which has been associated with increased dermal MC numbers in regressing AK lesions. Oyama et al. observed that AK lesions responding to imiquimod showed a significant rise in CD117^+^ MCs and proposed that these cells may support the anti-tumor immune response through TNF-β (Tumor Necrosis Factor beta) release [35]. In our study, PDT was associated with a marked reduction in MC infiltration in AK lesion. This difference may reflect the distinct biological effects of the two therapies, as imiquimod primarily stimulates local immune activation, whereas PDT is thought to act mainly through direct cytotoxicity and microvascular damage. At the same time, our data does not allow conclusions about whether the MCs remaining after PDT participate in wound healing, tissue remodeling, or angiogenesis. Likewise, although Rosin et al. suggested that activated MCs might contribute to reparative angiogenesis in incompletely regressed lesions, this interpretation should be considered hypothesis-generating rather than directly supported by our findings.

Furthermore, residual inflammatory and angiogenic activity after PDT may also reflect the involvement of tumor-associated macrophages (TAMs), although these cells were not specifically assessed in our study. As highly plastic cells within the tumor microenvironment, they may acquire either a proinflammatory, tumoricidal phenotype or a protumor, reparative phenotype, and this balance may affect the overall outcome of PDT [33,36,37,38,39]. The therapy itself may activate macrophages and enhance the release of reactive oxygen intermediates, TNF-α (Tumor Necrosis Factor alfa), and other inflammatory mediators, thereby supporting antitumor immunity; at the same time, these cells may also contribute to angiogenesis, tissue remodeling, and tumor repopulation in the later phase of the response [36,37]. This complexity is also relevant to MCs, which were the focus of our analysis. Beyond their direct effects on vascular and inflammatory remodeling, MCs may influence macrophage recruitment and polarization through paracrine mediators, including GM-CSF (granulocyte-macrophage colony-stimulating factor), CCL3 (macrophage inflammatory protein-1 α, MIP-1α), and IL-6 (interleukin-6) [33,38,39]. Accordingly, the post-treatment microenvironment likely reflects multidirectional crosstalk between these cell populations rather than a unidirectional immune response. Therefore, the reduction in MC infiltration observed in our study should be interpreted as part of a broader and still incompletely normalized stromal response after PDT.

Finally, another important consideration are Langerhans cells (LCs) which represent a key immunological component of the epidermis and may importantly modulate the biological effects of PDT in AK [40,41,42]. In human studies, topical ALA-PDT has been shown to decrease epidermal LCs density while inducing a migratory phenotype, indicating that their reduced epidermal presence reflects active trafficking rather than simple cell loss [40]. This observation is particularly relevant in the context of our results, because our study demonstrated that PDT also alters MC density within AK lesions, with a clear reduction after treatment but without complete normalization to the level of clinically healthy skin. Together, these findings suggest that PDT does not eliminate the local inflammatory microenvironment in a uniform manner but rather reshapes it in a cell-specific and spatially dependent fashion. The wound-healing literature supports this interpretation: after ALA-PDT, MCs have been shown to remain actively involved in tissue repair, including degranulation, close interaction with fibroblasts and dendritic cells, and participation in inflammatory and vascular remodeling [41]. In chronic wound models, MCs appear to function not merely as inflammatory effector cells, but as regulators of the reparative milieu, which is consistent with the partial persistence of MCs in our post-treatment AK specimens [41]. Therefore, the changes observed in MC count in our study are likely to reflect a broader PDT-induced remodeling of the cutaneous immune landscape, in which LCs, MCs, and TAMs may interact to influence both treatment response and the completeness of lesion clearance. The impact of PDT on these cell populations appears protocol-dependent and may vary according to the disease entity, photosensitizer, light dose, and treatment schedule.

Our study contributes to a broader understanding of tumor biology in the skin, particularly the early events in cutaneous squamous carcinogenesis. Angiogenesis is considered an important early process in the progression from AK to iSCC [18,33]. Nijsten et al. demonstrated that, as skin lesions progress from normal epidermis to AK and then to SCC, angiogenesis increases stepwise: their paired analysis showed significantly higher fractions of proliferating blood vessels in AK and Bowen’s disease (carcinoma in situ) than in to normal skin, with a further increase in iSCC [16]. These findings have been interpreted as supporting the concept that angiogenic changes may emerge at the premalignant stage. Angiogenesis provides the developing neoplasm with nutrients and growth signals and may facilitate the progression of incipient tumors beyond a minimal size. Our observation that AK lesions harbor dense microvasculature and MC infiltrates is consistent with this concept than early angiogenesis contributes to tumor progression.

Furthermore, the efficacy observed in our cohort should be interpreted in the context of baseline disease severity, the timing of post-treatment assessment, and the dynamic remodeling of the treated microenvironment. Although complete clinical clearance was achieved in 54.84% of patients and complete dermoscopic resolution in 25.81%, the overall treatment effect was substantially greater, as reflected by Olsen grade improvement in 93.5% of cases and the absence of persistent high-grade lesions (Olsen grade 2 or 3) after therapy. This indicates that PDT triggered a meaningful biological response, even in cases not meeting criteria for complete resolution. This is particularly relevant in the early post-treatment period, when ongoing tissue remodeling and immune-mediated regression may still be in progress. When compared with the evidence summarized in the European Dermatology Forum guideline by Morton et al. our complete-clearance rate appears lower, however, this difference is likely attributable to several methodological and biological factors [43]. In that guideline, c-PDT for AK is reported to yield typical lesion clearance rates of approximately 81–92% at around 3 months. By contrast, our evaluation was performed earlier, 8 weeks after the last treatment session, which may underestimate delayed clinical responses and ongoing post-PDT regression [43]. In addition, our cohort included a meaningful proportion of more advanced lesions at baseline, including Olsen grade 3 disease (25.8%), and more severe lesions are known to respond less completely to PDT. Thus, the lower apparent efficacy in our series most plausibly reflects a combination of earlier outcome assessment, greater baseline lesion burden, and lesion heterogeneity rather than an intrinsically lower treatment effect. These data also support the relevance of the local immune system and stromal compartment in shaping treatment response. In particular, LCs, TAMs, and MCs may contribute to both the immediate inflammatory reaction and the longer-term resolution phase after PDT. Their functional state, polarization, and spatial organization within the lesion and surrounding skin likely influence whether the post-treatment milieu favors durable clearance or persistent residual activity. From this perspective, incomplete clinical resolution does not necessarily indicate therapeutic failure but may instead reflect ongoing tissue remodeling and immune-mediated repair.

Summing up, our findings suggest that AK lesions are characterized by an altered inflammatory and vascular microenvironment. The increased MC count and MVD compared with clinically uninvolved skin, as well as their partial reduction after PDT, may be of exploratory interest when assessing tissue response. However, the persistence of elevated values after therapy and the MC–MVD correlation, which was stronger after PDT, should be interpreted cautiously and cannot be used to infer prognostic value or to guide clinical management.

## 5. Limitations

This paper has several limitations that should be acknowledged. Its small sample size, single-center design, and short follow-up period limit the generalizability of the findings and preclude any firm conclusions regarding long-term outcomes. In addition, the absence of an untreated, time-matched AK control must be considered when interpreting the results. Although the paired within-patient design reduced between-patient variability and clinically uninvolved skin provided a biological reference it cannot serve as a longitudinal control for the natural course of AK. The baseline biopsy may have initiated wound-healing and inflammatory processes that influenced subsequent MC count and MVD, while temporal variation, spontaneous regression, and regression to the mean may also have contributed to the observed changes. Therefore, the reductions in MC count and MVD should be interpreted as changes temporally associated with PDT rather than definitive evidence of direct PDT-induced microenvironmental modulation. The present data also does not permit inference regarding mast-cell activation, causality, or long-term prognostic significance.

## 6. Conclusions

To the best of our knowledge, this is the first study to prospectively assess the effect of PDT on MC-associated inflammatory activity and CD31-positive MVD in AK. In our cohort, PDT was associated with significant clinical and dermoscopic improvement, together with a marked reduction in MC infiltration and MVD. These findings indicate that the therapeutic effect of PDT extends beyond direct cytotoxicity toward dysplastic keratinocytes and is accompanied by remodeling of the local inflammatory and vascular microenvironment.

Although both parameters decreased after treatment, they remained higher than in clinically uninvolved skin, suggesting incomplete histological normalization despite clinical response. The persistent correlation between MC count and microvascular proliferation after PDT may be consistent with ongoing microenvironmental remodeling in chronically photodamaged skin. However, the present study does not allow conclusions regarding recurrence risk or prognostic significance.

Overall, MCs and angiogenesis appear to be relevant components of the AK microenvironment and may represent exploratory histopathological parameters associated with treatment response. Their clinical utility requires validation in larger multicenter studies with longer follow-up.

## Figures and Tables

**Table 1:** Inclusion and Exclusion Criteria for the Study.

**Inclusion Criteria**	
1	Signed informed consent for participation in the study.
2	Age ≥ 18 years.
3	Presence of a primary AK lesion with characteristic morphology, previously untreated.
4	Good general health, with no clinically significant conditions as assessed by the physician.
5	Ability to comprehend study-related information, adhere to protocol requirements, follow medical recommendations, and attend scheduled visits.
**Exclusion Criteria**	
1	Hypersensitivity to UVR or blue light.
2	Age < 18 years.
3	Pregnant or breastfeeding women, or those planning pregnancy during the study.
4	Participation in another clinical trial within 30 days prior to study initiation.
5	Inability or unwillingness to comply with study requirements.
6	Presence of acute inflammatory disease at the time of examination, with body temperature > 38 °C.
7	Active malignancy or history of cancer, including skin cancers (SCC, BCC, MM).
8	Autoimmune diseases, including Hashimoto’s thyroiditis, Graves’ disease, type 1 diabetes, rheumatoid arthritis, celiac disease, Crohn’s disease, systemic lupus erythematosus, dermatomyositis, polymyositis, ankylosing spondylitis, polymyalgia rheumatica, multiple sclerosis, Sjögren’s syndrome, and vitiligo.
9	Impaired wound healing.
10	Blood clotting disorders.
11	Chronic or intermittent use of NSAIDs during the study period.
12	Long-term anticoagulant therapy.
13	Long-term use of medications with known phototoxic effects.
14	Long-term immunosuppressive, immunomodulatory, or systemic corticosteroid therapy.
15	Planned hospitalization or surgical procedures during the study period.
16	Diastolic blood pressure > 95 mmHg or <65 mmHg.
17	Congenital or acquired immunodeficiency disorders.
18	Genophotodermatoses associated with increased skin cancer risk, such as xeroderma pigmentosum, Cockayne syndrome, and Bloom syndrome.
19	Renal dysfunction.
20	Psychiatric disorders with psychotic symptoms or major depressive disorder.
21	Substance abuse or dependence (alcohol, prescription medications, or illicit drugs) within the last 12 months.
22	Use of tanning beds or artificial UV exposure.

**Abbreviations:** AK, actinic keratosis; UVR, ultraviolet radiation; SCC, squamous cell carcinoma; BCC, basal cell carcinoma; MM, malignant melanoma; NSAIDs, non-steroidal anti-inflammatory drugs; UV, ultraviolet.

**Table 2:** The severity of actinic keratosis was evaluated using the clinical grading system proposed by Olsen [22] and a dermoscopic severity scale initially introduced by Zalaudek et al. [23]. Due to the presence of FC and the frequent coexistence of lesions at different stages within the same area, we introduced a modified version of the original dermoscopic scale. To improve the precision of grading, intermediate categories such as I/II and II/III were introduced, facilitating the classification of transitional AK lesions that did not fully meet the criteria for a distinct dermoscopic grade.

**Grade**	**Olsen’s Classification System**
1	lesions are slightly palpable, with the AK being more felt than visible
2	lesions are moderately thick, easily felt, and visible
3	lesions are very thick, hyperkeratotic, and/or clearly noticeable as AK
**Grade**	**Modified Zalaudek’s Dermoscopic Severity Scale**
I	red pseudo-network pattern with discrete white scales
I/II	features of I and II grade
II	an erythematous background, the so-called “strawberry pattern”
II/III	features of II and III grade
III	enlarged follicular openings with keratotic plugs over a scaly, white-to-yellow background

**Table 3:** Patient Characteristics. Baseline demographic and clinical characteristics of the study population prior to initiation of treatment (*n* = 31).

Characteristic	*n* (%)
Age (mean = 72.4 ± 8.64; range 54–92)	
≥72.4	18 (58.06)
<72.4	13 (41.94)
Sex	
Male	24 (77.42)
Female	7 (22.58)
Anatomic location	
Scalp	17 (54.84)
Face	9 (29.03)
Forearm	3 (9.68)
Trunk	2 (6.45)
Fitzpatrick Skin Type	
I	3 (9.68)
II	26 (83.87)
III	2 (6.45)
Olsen’s AK clinical grade	
1	12 (38.7)
2	11 (35.48)
3	8 (25.8)
Modified Zalaudek’s AK dermoscopic grade	
I	4 (12.9)
I/II	3 (9.68)
II	7 (25.81)
II/III	11 (35.48)
III	6 (19.35)

**Abbreviations:** *n*, number; AK, actinic keratosis.

**Table 4:** Pairwise comparisons of MC count and MVD among the three related tissue states.

Outcome and Comparison	Wilcoxon T	Raw *p*	Holm-Adjusted *p*	Median Paired Difference (95% CI)	Rank-Biserial Correlation (95% CI)
MC count: before PDT − control	0	9.313 × 10^−10^	2.794 × 10^−9^	14 (11 to 17)	1.000 (1.000 to 1.000)
MC count: after PDT − before PDT	0	9.313 × 10^−10^	2.794 × 10^−9^	−8 (−11 to −6)	−1.000 (−1.000 to −1.000)
MC count: after PDT − control	85	0.0008607	0.0008607	3 (1 to 5)	0.657 (0.341 to 0.905)
MVD: before PDT − control	0	9.313 × 10^−10^	2.794 × 10^−9^	15 (13 to 18)	1.000 (1.000 to 1.000)
MVD: after PDT − before PDT	8	1.788 × 10^−7^	3.576 × 10^−7^	−12 (−13 to −4)	−0.961 (−1.000 to −0.848)
MVD: after PDT − control	73	0.0005826	0.0005826	4 (2 to 7)	0.686 (0.361 to 0.931)

Wilcoxon T denotes the smaller sum of signed ranks. Holm’s procedure was applied separately to the three pairwise comparisons within each biomarker family. Confidence intervals were obtained using 10,000 patient-level bootstrap resamples. Overall Friedman-test results were χ^2^(2) = 52.323, *p* = 4.35 × 10^−12^, Kendall’s W = 0.844 for MC count and χ^2^(2) = 47.617, *p* = 4.57 × 10^−11^, Kendall’s W = 0.768 for MVD. **Abbreviations:** MC, mast cell; MVD, microvessel density; CI, confidence interval; PDT, photodynamic therapy.

## Data Availability

The data presented in this study is available on request from the corresponding author.

## Abbreviations

The following abbreviations are used in this manuscript:

AK	Actinic keratosis
cSCC	cutaneous Squamous Cell Carcinoma
UVR	Ultraviolet radiation
iSCC	invasive Squamous Cell Carcinoma
FC	Field cancerization
NMSCs	Non-Melanoma Skin Cancers
PDT	Photodynamic therapy
c-PDT	conventional PDT
dl-PDT	daylight PDT
5-ALA PDT	5-aminolevulinic acid-based PDT
5-ALA	5-aminolevulinic acid
MCs	Mast cells
CD31	Platelet Endothelial Cell Adhesion Molecule-1, PECAM-1
MC	Mast cell
MVD	Microvessel density
MM	Malignant melanoma
H&E	Hematoxylin and eosin
HPF	High-power fields
AEs	Adverse events
BCC	Basal Cell Carcinoma
VEGF	Vascular Endothelial Growth Factor
ECM	Extracellular matrix
Tregs	Regulatory T cells
TLR7	Toll-like receptor 7
TNF-β	Tumor Necrosis Factor beta
TAMs	Tumor-associated macrophages
TNF-α	Tumor Necrosis Factor alfa
GM-CSF	Granulocyte-macrophage colony-stimulating factor
CCL3	Macrophage inflammatory protein-1 alfa, MIP-1α
IL-6	Interleukin-6
LCs	Langerhans cells
